# Supplementary material for: Integrating and formatting biomedical data as pre-calculated knowledge graph embeddings in the Bioteque
Source: Nat Commun. 2022 Sep 9;13:5304. doi: 10.1038/s41467-022-33026-0 (PMC9463154; doi:10.1038/s41467-022-33026-0)
Supplement: Supplementary file 2 — Reporting Summary [file 41467_2022_33026_MOESM2_ESM.pdf]

nature portfolio

Corresponding author(s): Patrick Aloy

Last updated by author(s): 24/08/2022

Reporting Summary

Nature Portfolio wishes to improve the reproducibility of the work that we publish. This form provides structure for consistency and transparency in reporting. For further information on Nature Portfolio policies, see our Editorial Policies and the Editorial Policy Checklist.

Statistics

For all statistical analyses, confirm that the following items are present in the figure legend, table legend, main text, or Methods section.

n/a	Confirmed	
☐	☒	The exact sample size (n) for each experimental group/condition, given as a discrete number and unit of measurement
☐	☒	A statement on whether measurements were taken from distinct samples or whether the same sample was measured repeatedly
☐	☒	The statistical test(s) used AND whether they are one- or two-sided<br>*Only common tests should be described solely by name; describe more complex techniques in the Methods section.*
☒	☐	A description of all covariates tested
☐	☒	A description of any assumptions or corrections, such as tests of normality and adjustment for multiple comparisons
☐	☒	A full description of the statistical parameters including central tendency (e.g. means) or other basic estimates (e.g. regression coefficient) AND variation (e.g. standard deviation) or associated estimates of uncertainty (e.g. confidence intervals)
☐	☒	For null hypothesis testing, the test statistic (e.g. F, t, r) with confidence intervals, effect sizes, degrees of freedom and P value noted<br>*Give P values as exact values whenever suitable.*
☒	☐	For Bayesian analysis, information on the choice of priors and Markov chain Monte Carlo settings
☒	☐	For hierarchical and complex designs, identification of the appropriate level for tests and full reporting of outcomes
☐	☒	Estimates of effect sizes (e.g. Cohen's d, Pearson's r), indicating how they were calculated

Our web collection on statistics for biologists contains articles on many of the points above.

Software and code

Policy information about availability of computer code

Data collection	Custom code to collect the data used to generate the embedding resource is available from https://gitlabsbnb.irbbarcelona.org/bioteque
Data analysis	Data analysis has been performed using publicly available software, mostly based on Python (v 3.7). Statistical measures (e.g. Pearson correlations) and embedding distances were calculated using the SciPy package (v. 1.6.2.). Nearest neighbours rankings were obtained using the FAISS package (v. 1.6.5.). Machine learning model pre-processing, training and inference have been done using sklearn (v. 0.25) and xgboost (v. 1.3.3), optimized with hyperopt (v. 0.25). The Shap package (v. 0.38) was used to perform the Shapley analysis. The randomization of the HuRI-III network was done using pyBiRewire (v. 0.6), a python wrapper of the BiRewire Bioconductor package (https://github.com/andreagobbi/pyBiRewire). Hierarchical clustering on the HuRI-III heatmap matrix was done using the fascluster package (v. 1.1.26). ECFP4 fingerprints used in the repoDB prediction exercise were derived using the RDKit python package (v. 2020.09.1.0). Matplotlib (v. 3.4.2.) and Seaborn (v. 0.11.1) were used for figure generation. Wordclouds were generated using the wordcloud package (v. 1.8.1.). The circos plot was obtained using the circos software (v. 0.69-9, http://circos.ca/software/download/). 2D tSNE projections were obtained using the Multicore-opt-SNE package (https://github.com/omiq-ai/Multicore-opt-SNE).<br>To run node2vec, we used an optimized C++ implementation released for the metapath2vec algorithm, downloaded from: https://ericdongyx.github.io/metapath2vec/m2v.html.

For manuscripts utilizing custom algorithms or software that are central to the research but not yet described in published literature, software must be made available to editors and reviewers. We strongly encourage code deposition in a community repository (e.g. GitHub). See the Nature Portfolio guidelines for submitting code & software for further information.

Data

Policy information about availability of data

All manuscripts must include a data availability statement. This statement should provide the following information, where applicable:

- Accession codes, unique identifiers, or web links for publicly available datasets
- A description of any restrictions on data availability
- For clinical datasets or third party data, please ensure that the statement adheres to our policy

The metapath embeddings used in the manuscript can be downloaded from the Bioteque resource (https://bioteque.irbbarcelona.org/downloads). Accessible links to all the sources of the datasets embedded in the Bioteque resource are listed on https://bioteque.irbbarcelona.org/sources. RMA normalized expression data from GDSC cell lines was downloaded from https://www.cancerrxgene.org/gdsc1000/GDSC1000_WebResources/Home.html. CCLE RNAseq data was downloaded from https://sites.broadinstitute.org/ccle/datasets. Cell line tissue of origin annotations were obtained from clue.io (https://clue.io/cell-app). The HuRI-III network was downloaded from http://www.interactome-atlas.org/download. The first release (v1) of repoDB indications was downloaded from http://apps.chiragjpgroup.org/repoDB/. The second release (v2) of repoDB indications was downloaded from https://unmtid-shinyapps.net/shiny/repodb/. ATC codes were obtained from DrugBank (https://go.drugbank.com/releases/latest#full), DrugCentral (https://drugcentral.org/download), and KEGG (https://www.genome.jp/kegg-bin/get_htext?br08303+D00731). Curated gene-disease associations were downloaded from DisGeNET (https://www.disgenet.org/downloads).

Field-specific reporting

Please select the one below that is the best fit for your research. If you are not sure, read the appropriate sections before making your selection.

☒ Life sciences ☐ Behavioural & social sciences ☐ Ecological, evolutionary & environmental sciences

For a reference copy of the document with all sections, see nature.com/documents/nr-reporting-summary-flat.pdf

Life sciences study design

All studies must disclose on these points even when the disclosure is negative.

Sample size	No sample size was calculated. Instead, we used all the data available in the data repositories. Notice that these repositories have been thoroughly analyzed by the original authors (properly cited in the text) and most of them have been extensively exploited in downstream applications. Importantly, quality filters were implemented to assess the metapath embeddings, thus excluding from our resources those datasets that lead to not robust spaces (see Data exclusions).
Data exclusions	Entities not included in the universe predefined by the used vocabularies were not included in the knowledge graph. The data universe for each entity type is provided in Supplementary Data 1. Additional filtering cutoffs used during the pre-processing of each dataset in the Bioteque are specified in the corresponding dataset pre-process links on https://bioteque.irbbarcelona.org/sources.<br>Embedding metapaths that did not fulfill the imposed quality requirements (explained in the text) were removed from our resource. A list of these metapaths can be found in Supplementary Data 3, together with an explanation justifying their removal.<br>Finally, in the drug-disease prediction exercise, some drug and disease terms were pruned to avoid biases in the training of the model. The pruning was solely based on statistical measurements as explained in the Methods section. The pruned terms are specified in Supplementary Figure 5.
Replication	To assess the robustness of the embedding pipeline we randomly selected 10 metapaths of the resource and run the embedding procedure 10 times, verifying that the embedding spaces were comparable in terms of network preservation (i.e. preservation of neighbours) and recapitulation of orthogonal associations.
Randomization	For each drug model in the GDSC drug response prediction, we used a stratified 10 cross-validation (using the 'StratifiedKFold' method in sklearn, v. 0.25) to allocate the cell line samples in train-test splits. In the prediction of HuRI-III, we used a stratified 20 cross-validation (using the same 'StratifiedKFold' method) to allocate samples in train-test splits. In the prediction of repoDB indications, all compound-disease samples available in the first release (repoDB v1) were allocated in the train set, while new samples included in the second release (repoDB v2) were allocated in the test set. Samples in the train set were randomly shuffled before training the model.
Blinding	All data splits used for the training of the models have been randomly shuffled and run through the computational pipeline in a blind way. The corresponding split strategies varied depending on the task and, accordingly, are properly described in the pertinent sections within the Methods.

Reporting for specific materials, systems and methods

We require information from authors about some types of materials, experimental systems and methods used in many studies. Here, indicate whether each material, system or method listed is relevant to your study. If you are not sure if a list item applies to your research, read the appropriate section before selecting a response.

Materials & experimental systems

n/a	Involved in the study
☒	☐ Antibodies
☒	☐ Eukaryotic cell lines
☒	☐ Palaeontology and archaeology
☒	☐ Animals and other organisms
☒	☐ Human research participants
☒	☐ Clinical data
☒	☐ Dual use research of concern

Methods

n/a	Involved in the study
☒	☐ ChIP-seq
☒	☐ Flow cytometry
☒	☐ MRI-based neuroimaging
