# Supplementary material for: Integrating and formatting biomedical data as pre-calculated knowledge graph embeddings in the Bioteque
Source: Nat Commun. 2022 Sep 9;13:5304. doi: 10.1038/s41467-022-33026-0 (PMC9463154; doi:10.1038/s41467-022-33026-0)
Supplement: Supplementary file 4 — Description of Additional Supplementary Files [file 41467_2022_33026_MOESM4_ESM.pdf]

Title: Supplementary Data 1.
Description: Nodes universe considered in the resource for each of the embedded entities. The node name together with some metadata are provided along the identifier. The ‘pruned general terms’ tab contains those entities that were removed from ontologies.

Title: Supplementary Data 2.
Description: Description of the associations between nodes (i.e. metapaths) and the datasets accomodated in the knowledge graph (KG).

Title: Supplementary Data 3.
Description: List of all the metapath embeddings available in the Bioteque resource. We also list those metapath that were removed from the resource for being supoptimal.
